# Analysis of mass spectrometry data using sub-spectra

**DOI:** 10.1186/1471-2105-10-S1-S51

**Published:** 2009-01-30

**Authors:** Wouter Meuleman, Judith YMN Engwegen, Marie-Christine W Gast, Lodewyk FA Wessels, Marcel JT Reinders

**Affiliations:** 1 Bioinformatics and Statistics, Department of Molecular Biology, The Netherlands Cancer Institute, Amsterdam, The Netherlands; 2 Information and Communication Theory Group, Faculty of Electrical Engineering, Mathematics and Computer Science, Delft University of Technology, Delft, The Netherlands; 3 Department of Pharmacy & Pharmacology, The Netherlands Cancer Institute/Slotervaart Hospital, Amsterdam, The Netherlands

## Abstract

**Background:**

Spectra resulting from Surface-Enhanced Laser Desorption/Ionisation (SELDI) mass spectrometry measurements are constructed by combining sub-spectra, each of which are the result of a single firing of the laser responsible for the process of desorption/ionisation. These firings are performed at different locations of the spot on which the sample is analysed. The final spectrum is then constructed by summing over all these sub-spectra. This process is sub-optimal in that it can average out peaks from peptides that are present in low abundance or are unevenly distributed across the spot, particularly because the amount of noise varies considerably between sub-spectra. This argues for analysing sub-spectra separately and combining results afterwards.

**Results:**

Here, we propose to analyse these sub-spectra one-by-one and combine the results using a framework which includes a significance test. This allows one to, for the first time, attach a confidence measure to detected peaks, based on the signal strength of a peak across sub-spectra. In a comparison with three other approaches the sub-spectral approach achieves a higher sensitivity and a low FDR. We further introduce the notion of peak-bags, which provide rich information about the sub-spectral contributions to a given peak.

**Conclusion:**

The proposed procedure offers better control over the process of distinguishing signal from noise, resulting in an improved performance over other available methods. Moreover, our method provides an implicit deconvolution of peaks, yielding insight in the actual shape of a peak, potentially aiding in a deeper understanding of peak distribution.

**Availability:**

Implementations of the algorithm in R are available upon request.

## Background

Surface-Enhanced Laser Desorption/Ionisation (SELDI) Time-Of-Flight (TOF) mass spectrometry [1] allows one to scan the (sub-)proteome of a biological sample. The sample, e.g., purified serum, is applied to a spot on a chip and repeatedly irradiated by a laser, which causes peptides contained in the sample to desorb from the spot and become ionised (charged), which is crucial for the subsequent process of mass-separation and detection.

The used laser beam has, depending on the machine model employed, either an elliptical or round shape. In any case, its size does not allow for a full coverage of the complete spot in one go. Therefore, in order to cover most of it, the laser probes different positions of the spot, resulting in *sub-spectra *(also termed *single-shot spectra *or *transients*) for each location. By default, a final spectrum is constructed by summing over all sub-spectra, which is then presented to the user.

The individual sub-spectra however, contain a wealth of information, that is normally missed by studying full spectra only. This includes information on spatial differences between sub-spectra, such as the total protein and matrix material content and the amount of noise, which all can vary considerably between sub-spectra due to, e.g., inhomogeneity of the sample and various experimental factors.

**Figure 1:**
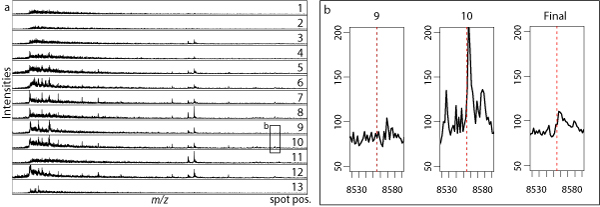
**Examples of sub-spectra**. (a) Example of differences in the amount of signal and noise in sub-spectra resulting from measurements at different spot positions. The spot positions are indicated on the right. (b) Examples of a peak corresponding to ubiquitin (mass indicated by red dotted line) in two sub-spectra and the full (aggregated) spectrum.

Figure 1a shows sub-spectra of an example spectrum, displaying large global differences in the amount of signal and noise between spot positions.

This is made more clear in Figure 1b. The first two panels show sub-spectra at spot positions 9 and 10 for the mass region corresponding to the ubiquitin protein. This clearly shows the large possible differences in signal between spot positions. The third panel shows the full spectrum, resulting from averaging over all sub-spectra. Indeed, here the ubiquitin peak is not significantly higher than the background.

In case of an inhomogeneous distribution of peptides over the spot, for example in the case of a low abundant peptide, taking the mean over all sub-spectra with these highly varying signal and noise levels can average out peaks, causing only the most abundant peptides to appear in the final spectrum. In Additional file 1, section 1, we describe a simple experiment that simulates this behaviour, showing that for arbitrary signals with varying noise levels it is indeed beneficial to study them separately.

We are not alone [2] in believing that the default data acquisition process is sub-optimal and that it is beneficial to analyse individual sub-spectra and combining findings afterwards. Sköld *et al. *have also analysed sub-spectra before, mainly within the scope of imputing missing values, i.e., identifying and recovering saturated spectral peaks. Our focus is on peak detection. More specifically, by analysing individual sub-spectra and combining results afterwards, we account for differences in noise levels between spot positions, decreasing the chance of losing peaks from peptides that are present in low abundance or are unevenly distributed over the spot.

Furthermore, we are able to quantify the confidence of detected peaks being true positives. The analysis of sub-spectra allows us to test the significance of peaks based on their amplitude in these sub-spectra, largely eliminating the need for (arbitrary) parameter settings during the peak detection process.

## Results and discussion

The approach we take involves a low-level analysis of individual sub-spectra using wavelets, followed by a method to assess the significance of peaks detected in the sub-spectra. We show that our method compares favourably to a number of existing methods by using spectra resulting from a carefully designed spiking experiment. Furthermore, we show that our method offers an implicit deconvolution of peaks through the notion of *peak-bags*.

### Analysis of sub-spectra

SELDI (sub-)spectra exhibit much noise, including a strong baseline effect caused by the use of energy absorbing matrix material inherent to the technology. Conventionally, this baseline effect is estimated and subtracted from the data explicitly, using *ad hoc *baseline correction algorithms. Methods based on wavelets [3-5] model this noise implicitly, assuming its frequency and shape is fundamentally different from that of the signal.

We use the algorithm proposed by Du *et al. *[4] to detect peaks in *individual sub-spectra*. They use an approach based on a continuous wavelet transform. By scaling and translating a so-called mother wavelet function, a spectrum is decomposed into a 'scale space', where low scales model high frequency noise and high scales model low frequency global trends in the data. Peaks can be found by identifying ridges in this scale space, corresponding to regions where the spectrum is highly correlated with the wavelet function.

This process is described in more detail in the Methods section ('Wavelet analysis on full spectrum'). Although we consider the scale space approach taken by Du *et al. *to be elegant, their algorithm employs a number of fairly arbitrarily chosen parameters to identify ridges as being peaks. For instance, the range and number of scales across which ridges are detected and a signal-to-noise ratio threshold. Instead, we consider all ridges detected in sub-spectra to be candidate peaks and use them as input to subsequent analyses, regardless of these parameters.

### Peak significance

**Figure 2:**
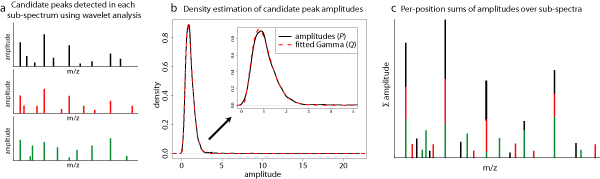
**Combined analysis of sub-spectra**. (a) Peaks detected in each sub-spectrum using the wavelet analysis. We obtain one set of peaks for each sub-spectrum, although in this cartoon we show only three, for simplicity. (b) Using the distribution of *all *peaks detected across *all *sub-spectra (*P*, solid line), we estimate a distribution of noise peaks (*Q*, dashed line). (c) Sets of peaks obtained in (a) are summed per *m/z *position and used as input for the significance test.

Instead of using parameter-based peak-detection, we rely on a significance test for candidate peaks. The wavelet analysis yields, for each sub-spectrum, a set of identified (candidate) peak positions and amplitudes in the form of peak signal-to-noise ratios (Figure 2a). All these single-peak amplitudes, i.e., from all sub-spectra, form a peak distribution *P *which roughly follows a Gamma distribution (Figure 2b). This distribution consists of both real peaks as well as a background distribution. In order to estimate the background distribution, we use an iterative procedure of removing high values, i.e., 'real' peaks, and re-fitting a Gamma distribution, until we obtain a distribution *Q *of 'noise' peaks (see Methods section ('Estimating the noise distribution')).

In order to assess peak significance, we aggregate the wavelet analysis results from all sub-spectra. To obtain the aggregate spectrum, we sum the sub-spectral values per *m/z *value across all sub-spectra (Figure 2c). The significance of one particular *m/z *position is assessed by comparing the value in the summed peak spectrum to a null distribution, yielding a p-value. This null distribution is the probability density function associated with the random variable which is obtained by summing *n *(number of sub-spectra) random variables distributed according to *Q*. In other words, this null distribution is a *summed sampling distribution *of *Q*, denoted *Q*_Σ_. Please refer to the Methods section ('Peak significance test') for a more elaborate discussion on this procedure.

Mass spectrometers suffer from a limited mass accuracy. To account for small deviations in peak positions across sub-spectra, we extend the significance test to multiple *m/z *positions simultaneously, i.e., all positions contained in a sliding window of size *w*. For multiple window sizes, this results in a second scale space of p-values with the *m/z *positions of the window on the horizontal axis and the window size on the vertical axis. Each position contains the p-value of the test result at that *m/z *position with the given window size. All p-values are corrected for multiple testing per window size (i.e., row-wise) over all non-empty windows using the procedure proposed by Benjamini and Hochberg [6].

In this second scale space, we now search for clusters containing as many *m/z *positions as possible that are significant *and *have at most one contribution from each sub-spectrum. The latter is used to prevent finding clusters containing peaks for actually different peptides; these should result in separate clusters. We start the search from the most significant peak at a minimal window size. In an iterative process, the window size is gradually increased, up to the point where either

• the combined values of peaks within the window are not significant anymore, or

• we include more than one contribution from a single sub-spectrum.

The results of this procedure we call *peak-bags *and they encompass a central peak position, a range of *m/z *values in a window centered around this peak position, and a signal level for each *m/z *value, also registering which sub-spectra contributed to this signal. The signal level is a summation of the wavelet coefficient values for the contributing sub-spectra.

**Figure 3:**
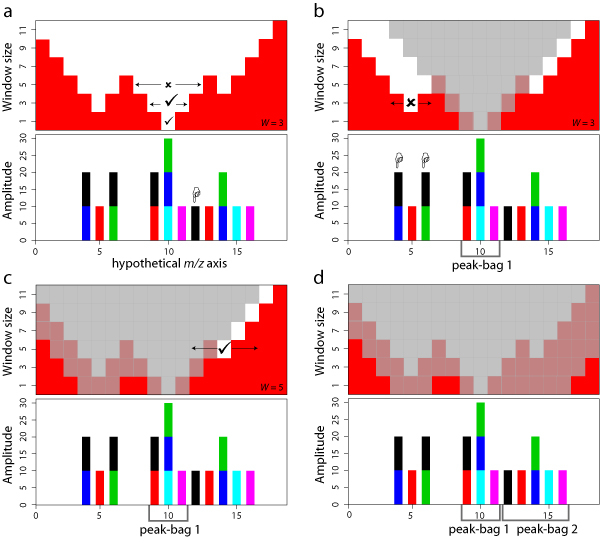
**Cartoon depicting the process used to identifypeak-bags**. Each panel shows the same example wavelet analysis output (bottom), with peaks coloured corresponding to the originating sub-spectra, and a hypothetical p-value scale space (top), where white regions are termed significant. (a) Analysis over multiple window sizes. Window size 5 at *m/z *= 10 is significant, but has multiple contributions from the same sub-spectrum (i.e., the black peaks). Therefore, a window of size 3 is used for the new peak-bag, since it is the largest significant window size with at most one contribution from each sub-spectra. Scale space elements that, when used as a future window-center, reference peak positions lying in the current window, are greyed out and the process restarts. (b) Although the combined peaks are significant, there are again multiple contributions from one sub-spectrum at window size 3. Position 5 at window size 1 is not significant. (c) Peak-bag at slightly higher scale. Larger windows are greyed out, so a new peak-bag is selected with window size 5. (d) No significant elements remaining.

Figure 3 contains a cartoon of the iterative process to detect peak-bags. In Panel (a), the position that is significant at a minimal window size is Position 10 at window size 1. At a window size of 3, this is still significant. Also, the positions within this window (Positions 9, 10 and 11) contain at most one contribution from each sub-spectrum. At a window size of 5 however, a second 'black' peak is included (Position 12), thereby violating the rules. Therefore, the window of size 3, centered on Position 10, is selected as a new peak-bag. In Panel (b), the scale space elements that will not be used for detecting future peak-bags are greyed out. These elements are the ones that, when used as a future window-center, would reference peak positions already belonging the newly identified peak-bag. More formally, for each window size *w *in the scale space, with *x*_*i *_being the center of the new peak-bag, this includes positions *x*_*i *_- ((*w*-1)/2) - ((*W*-1)/2) to *x*_*i*_+((*w*-1)/2)+((*W*-1)/2), where *W *is the window size of the new peak-bag.

The next significant position is Position 5 (window size 3). However, this has two contributions from the same sub-spectrum (the black peaks). Position 5 alone (i.e., at window size 1) is not significant. Therefore, no new peak-bag is defined. However, in Panel (c) the appropriate elements in the significance scale space are still greyed out. This is to avoid re-analysis of Position 5. Position 14 at window size 5 is significant, contains only single contributions and no larger window exists. It is selected as a second peak-bag. Panel (d) shows the result of greying out the appropriate elements again. No significant elements are remaining, finalising the analysis.

### Experiments

We performed SELDI-TOF mass spectrometry twice on 16 samples containing a mixture of 5 spiking peptides. This way, we obtained 32 full spectra and their respective sub-spectra. The spiking peptides used are dynorphin (2147.5 Da), ACTH 1–24 (2933.5 Da), *β*-endorphin (3465.6 Da), insulin (cow pancreas; 5733.6 Da) and ubiquitin (8564.8 Da).

We employed our sub-spectral analysis method and compared the obtained peak-bags to the results of three methods for analysing full mass spectra. These are the wavelet-based method proposed by Du *et al. *[4], a standard method implemented in the PROcess R-package and another method implemented in the MASDA R-package. MASDA implements an elementary SELDI-TOF analysis pipeline, used for the comparison of normalisation methods [7]. Except for the approach presented here, all these methods employ a signal-to-noise ratio cutoff in order to detect peaks.

**Figure 4:**
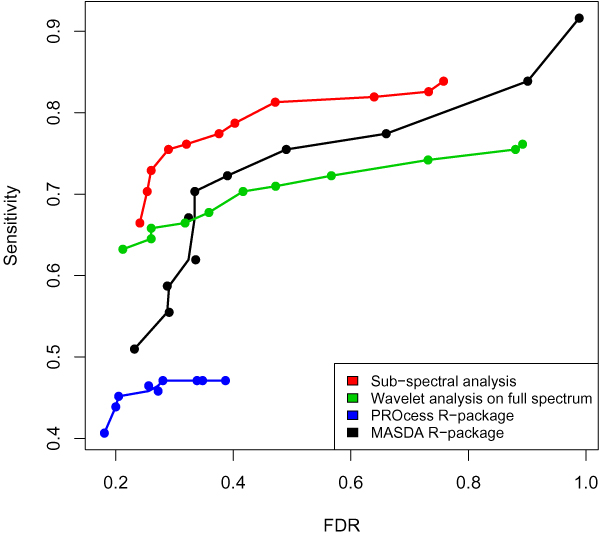
**Operating Characteristic curves**. Operating Characteristic (OC) curves evaluating the performance of four analysis methods, including the method proposed here.

For the comparison of results, we assessed the sensitivity and False Discovery Rate (FDR) with respect to finding peaks corresponding to the five spiking peptides. By varying the parameters that influence the number of detected peaks (p-value or signal-to-noise ratio) over a wide range of values, and computing the sensitivity and FDR at every setting, we can construct and Operating Characteristic (OC) curve (see Figure 4).

Ideally, the sensitivity of a method should be as high as possible, while keeping the FDR as small as possible. In other words, we would like to be as much as possible towards the topleft position of the graph in Figure 4.

The sub-spectral analysis clearly outperforms the other (full spectrum based) methods by achieving both a high sensitivity and low FDR across a relatively large range of p-value thresholds. Even when the FDR is high, the sensitivity of the sub-spectral analysis clearly outperforms all other approaches. Although it has been shown in the literature [4,5] that the wavelet-based analysis often outperforms the traditional analysis methods, such as the one implemented in the MASDA package, here it only seems to outperform the latter in the low FDR range. This is, however, not so bad, as in most proteomics analyses it is actually desirable to keep the FDR at a low level. The PROcess package generally performs the worst. Although it displays the lowest FDR, its sensitivity is extremely low.

### Peak-bags

**Figure 5:**
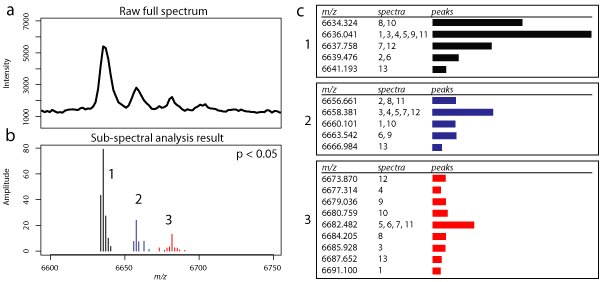
**Examples of peak-bags found by our algorithm in a spectrum of human serum**. (a) Full spectrum (b) Identified peak-bags with *p *< 0.05. (c) Details of peak-bags with *m/z *values, contributing sub-spectra and a graphical representation of the peaks in the bag.

The methods we compare our approach with do not provide any information on the shape of peaks. Our method, based on analysing sub-spectra, yields peak-bags that do provide this information, as well as a confidence measure in the form of a p-value. Figure 5 shows part of a spectrum of a human serum sample (full spectrum is shown in Additional file 1, section 5) with one large peak and what seems to be several adduct peaks of the same peptide.

The methods implemented by Du *et al. *and the PROcess package find only one peak here, namely the largest one. The MASDA package finds some of the adduct peaks as well, albeit at an unrealisticly low signal-to-noise ratio cutoff. Sub-spectral analysis however, yields multiple peak-bags. In the figure, each colour represents a peak-bag with its associated peak positions and amplitudes. Note that these peak-bags are selected with *p *< 0.05 and have at most one contribution from each sub-spectrum, as shown in Figure 5.

## Conclusion

We have shown that analysing sub-spectra allows one to find real peaks not found by other methods. Our results are not heavily dependent on parameter settings, such as a signal-to-noise ratio threshold for detecting peaks. Instead, for the first time, we provide a confidence measure for peaks in the form of p-values, reducing the false positive rate and yielding a better sensitivity.

Furthermore, our notion of peak-bags provides information on the variability and distribution of peaks across sub-spectra and their contribution to the aggregate (full) spectrum. This provides a more complete representation of peaks, impossible to obtain using full spectra. Effectively, our approach offers an implicit way of deconvoluting spectra.

## Methods

### Sample pre-processing

Spiking mixture was freshly prepared from individual peptides (Ciphergen Biosystems Inc., Freemont, CA, USA). A 100 *μl*-stock solution containing a mixture of dynorphin (2147.5 Da), ACTH 1–24 (2933.5 Da), *β*-endorphin (3465.6 Da), insulin (cow pancreas; 5733.6 Da) and ubiquitin (8564.8 Da), each 1 nmol/100 *μl*, in deionised water was prepared. In pilot experiments the optimal dilution for spiking in matrix was assessed, resulting in an optimal dilution of 1:1000 when applying 20 *μl *of sample and 2 *μl *of matrix to the chip.

Before the experiment, the serum sample was thawed and denatured by adding 190 *μl *of a solution of 9 M urea and 2% CHAPS, (Sigma, St. Louis, MO, USA) to 10 *μl *of serum. As energy absorbing matrix a 50% solution of sinapinic acid (SPA; Ciphergen Biosystems) in 50% acetonitrile (ACN) + 0.5% trifluoracetic acid (TFA) was used. Spiked matrix was prepared by adding 6 *μl *of a 1:15 dilution of spiking solution to a total of 400 *μl *SPA solution.

We performed SELDI-TOF mass spectrometry (Ciphergen Biosystems) with CM10 chips (weak cation exchange chip containing anionic carboxylate groups that bind positively charged proteins in serum) with a 100 mM sodium acetate (Sigma) binding buffer, pH 4, and a 50 mM HEPES wash buffer.

During all steps of the protocol, the bioprocessor was placed on a platform shaker at 350 rpm. Chips were equilibrated twice with 200 *μl *of binding buffer for 5 min. Subsequently, 180 *μl *of binding buffer and 20 *μl *of denatured sample were applied to the chip surface. Incubation was set to 30 min. After binding, the chips were washed twice for 5 min with binding buffer, followed by two 5-min washes with wash buffer. Lastly, chips were rinsed with deionised water, air-dried and finished with two 1-*μl *SPA applications to the sample spots.

### Data acquisition

Protein chips were analysed using the PBS-IIC ProteinChip Reader (Ciphergen Biosystems). Data were collected between 0 and 100000 Da, optimisation range from 1500 to 50000 Da, laser intensity 155, detector sensitivity 5 and laser focusing at 10000 Da. We probed spot positions 20 to 80, with intervals of 5 and with five repeat shots per position, yielding in total 65 sub-spectra per spot. For information on how to obtain sub-spectra, please refer to Additional file 1, section 6.

We performed a two-way ANOVA analysis, with 'spot position' and 'repeat shot index' as covariates, to test whether there are significant differences in noise levels that can be explained by either one of these covariates. We confirmed the findings of [2] in the sense that in general we find a significant portion of the variance to be explained by spot position and not by shot index. This lead us to the decision to sum sub-spectra on a per-position basis, thereby reducing the computational complexity of the study, while still enabling us to show the potential of our method. This procedure leads to the 13 sub-spectra described in the paper.

### Full spectrum approaches

#### PROcess R-package

We use the PROcess R-package exactly as recommended in the accompanying documentation (i.e., the R *vignette*). The peak discriminating parameter we vary for the performance assessment is the signal-to-noise ratio.

#### MASDA R-package

An often used method of analysing a mass spectrum is by extracting the (monotonically decreasing) baseline signal, normalising the spectrum and detecting peaks above a certain pre-specified noise level. Normalisation is done here using a method found to perform well in an earlier study [7], namely by extracting the mean from a spectrum and subsequently dividing it by the standard deviation. Peaks are selected above a threshold (the signal-to-noise ratio), defined by the mean plus a number of times the standard devation, both of which are estimated within a local window of 1000 measurement points. These procedures are implemented in the MASDA R-package for mass spectrometry data analysis, freely available from [8].

#### Wavelet analysis on full spectrum

Following [4], we employ a *mexican hat *mother wavelet, which is proportional to the second derivative of a Gaussian function and is similar in shape to actual peaks found in a mass spectrum.

The mother wavelet is scaled and translated over spectra, during which the inner product between the wavelet and the signal is calculated, resulting in a scale space of wavelet coefficients. This scale space is used to extract features, i.e., peaks, from spectra.

*Ridges *in this space are identified, by locating local maxima over scales. Du *et al. *then filter these according to a certain parameter set, consisting of, among others, the range of scales in which peaks are detected, the minimal length of identified ridges and a signal-to-noise ratio threshold. The *m/z *axis locations of the ridges that pass these filtering steps they label as peak positions. We used the default parameters, such as used in [4]. Additional file 1 (section 2) contains an illustration of this analyis.

### Analysis of sub-spectra

To analyse sub-spectra, we used the same procedure as for full spectra, except that we do not employ the parameters proposed by Du *et al.*. We do not filter ridges at this point to distinguish 'real' peaks from 'noise' peaks. Rather, we retain information on all identified ridges, in order to construct a noise distribution.

#### Estimating the noise distribution

We have confirmed that the distribution of wavelet coefficients for an empty spectrum approximately follows a Gamma distribution (see Additional file 1, section 3). In order to estimate the noise distribution *Q*, we iterate over the following procedure:

1. *Q' *= *P*

2. Remove upper 0.01% quantile values from *Q'*

3. Fit a Gamma distribution to *Q'*

4. Assess maximum squared error (MSE) between fitted Gamma and *Q'*

5. Go to step 2

The Gamma for which the MSE is lowest, i.e., only containing 'noise' peaks, is selected as the noise distribution *Q*. Please also refer to Additional file 1 (section 4) for more information on this. The detection of peaks then follows the procedure as described in the main text, and peak-bags are constructed using a significance threshold (p-value).

#### Peak significance test

The wavelet analysis results for a random, noise-only, spectrum can effectively be seen as a sampling from the estimated distribution *Q *~ Γ (*k*, *θ*). Per sub-spectrum and *m/z *position, one value is drawn from *Q*. For *n *acquired sub-spectra, *n *values are drawn. The value for one *m/z *position in a full (summed) spectrum is therefore approximately the sum of these *n *values. The random variable representing this sum follows a *summed sampling distribution*, *Q*_Σ_, of *n*-summed values drawn from *Q*. It follows from the Central Limit Theorem that for large enough *n*, the distribution of *Q*_Σ _approximates a Normal distribution, defined by *N*(*nkθ*, *nkθ*^2^), where *kθ *and *kθ*^2 ^are the mean and variance of *Q*, respectively. For one particular *m/z *position, the significance can be assessed by comparing the summed signal of all *s *peaks at that position, plus *n *– *s *times the mean of *Q *(i.e., *kθ*), against *Q*_Σ_. For multiple *m/z *positions, i.e., in a sliding window of size *w*, a summed sampling distribution is used where *n *is multiplied by the window size *w *and *s *is equal to the number of peaks in the window.

### OC curve construction

We assessed the performances of the different peak detection methods using Operating Characteristic curves. Such curves are similar to Receiver Operating Characteristics curves, which register the trade-off between sensitivity and specificity. OC curves however, utilise the False Discovery Rate instead of specificity. This is much more useful in the context of mass spectrometry data, as the presence of real and noisy peaks is extremely unbalanced, in favour of the latter.

OC curves register the trade-off between sensitivity (or True Positive Rate, TPR) and False Discovery Rate (FDR), defined as follows:

$$\begin{array}{lll} \text{TPR} & = & \text{TP}/\text{P}\\ & = & \text{TP}/(\text{TP}+\text{FN})\\ \text{FDR} & = & \text{FP}/(\text{FP}+\text{TP}). \end{array}$$

Here, the set of positives (P) consists of all peaks detected by a method in one run. True positives are peaks corresponding to the mass of the five spiked-in peptides described earlier. A peak is called a true positive if it lies within 0.2% [9-11] of the mass of one of these peptides plus one proton [12]. False negatives are the true positive peaks not found by a method. False positives are peaks detected by a method that do not correspond to any one of the spiked-in peptides.

In order to construct an OC curve, results are obtained for each method while varying a parameter value that determines the number of peaks detected by that method. This threshold is the p-value resulting from the significance test for the sub-spectra based method and the signal-to-noise ratio for all other methods. Results from the 32 spectra assessed are averaged per threshold value and plotted. A line is fitted through these points using a local running median function.

## Competing interests

The authors declare that they have no competing interests.

## Authors' contributions

WM performed the analyses. WM, MJTR and LFAW designed the algorithms and dry-lab experiments and wrote the manuscript. WM, JYMNE and MCWG designed and performed the wet-lab experiment with the spiking peptides. All authors read and approved the final manuscript.

## Supplementary Material

Additional file 1 **Additional details**. File containing additional details about analysis process. For now available from: . Click here for file
