# Supplementary material for: Analysis of mass spectrometry data using sub-spectra
Source: BMC Bioinformatics. 2009 Jan 30;10(Suppl 1):S51. doi: 10.1186/1471-2105-10-S1-S51 (PMC2648731; doi:10.1186/1471-2105-10-S1-S51)
Supplement: Additional file 1 — Additional details. File containing additional details about analysis process. For now available from: . [file 1471-2105-10-S1-S51-S1.pdf]

Supplementary material

Contents

1. Simulation experiment
2. Illustration of wavelet analysis
3. Estimation of ‘peak’ distribution in an virtually empty (matrix only) spectrum
4. Estimation of Q from P
5. Example of spectrum of spiked human serum
6. Procedure to obtain sub-spectra

1. Simulation experiment

We generated 13 signals under two models. In the first model the statistical characteristics of the background, i.e., mean and standard deviation, are all similar, while in the second model the characteristics vary between the signals.

More specifically, the signals were drawn from Gaussian distributions. We make a distinction between signals with constant characteristics and signals with varying characteristics. For the former, we sampled the signals from a Gaussian distribution with parameters $\mu = 75$ and $\sigma = 10$. For the latter, we used $\mu \sim N(75,5)$ and $\sigma \sim N(10,5)$. These parameters have been derived from real sub-spectra, by using robust estimators of their background noise, even though we are not really simulating sub-spectra here, but just normally distributed random signals.

The 13 generated signals are used as input to the simulation experiments. In these experiments, we add an artificial peak of a particular height at a fixed pre-defined position to a number of signals. The height of the peak (*x* times the standard deviation of the distribution used to generate the background) and the number of signals in which the peak appears are varied from 1 to 10 and from 1 to 13, respectively.

For each of the combinations of peak amplitude and number of peak-containing signals, we can now investigate which of the following two methods yields the highest signal-to-noise ratio for the peak position:

1. Sum all 13 signals, apply a mean-variance normalisation and return the peak amplitude, i.e., the signal-to-noise ratio. This would be a very simple model for full spectrum analysis. More formally:

$$\mathrm{SNR}\left(\sum_{i=1}^{13} S_i\right)$$

where S_i indicates one of the 13 sub-spectra and SNR() is a function that normalises a signals and returns
the peak amplitude, i.e., effectively the signal-to-noise ratio of the peak.

2. Apply a mean-variance normalisation to each individual signals, obtain the peak amplitudes and return the mean of these; a model for sub-spectral analysis. More formally:

$$\frac{1}{13}\sum_{i=1}^{13} \mathrm{SNR}\left(S_i\right)$$

This whole procedure is performed separately for the two types of data, i.e., with constant or varying characteristics between signals, and is repeated 100 times. Figure S1 depicts the mean improvement in detected signal-to-noise ratio of Method 1 over Method 2 for these simulation experiments. From this figure, we see that signals with varying characteristics benefit greatly from per-signal (i.e., sub-spectral) analysis in terms of improvement of signal-to-noise ratios of peaks, unlike signals with constant characteristics.

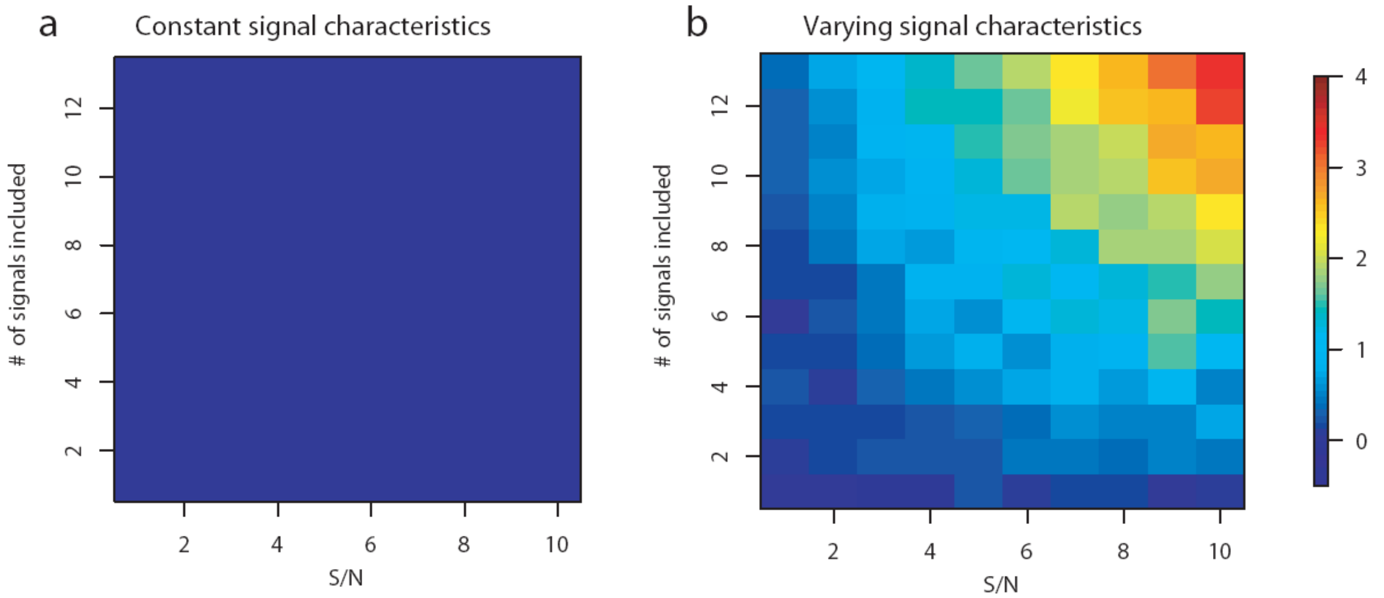

Figure S1: Simulation results to study the advantage of analysing sub-spectra over full spectra. For this simulation, 13 signals are generated by sampling from a Gaussian distribution and adding a true peak of varying amplitude to a fixed known position in a subset of signals. The signal-to-noise ratios of the real peaks are varied from 1 to 10 and the number of signals containing a true peak was varied from 1 to 13. The figure shows the mean improvement in detected signal-to-noise ratio of peaks over 100 repeat runs. We compare scenarios in which the characteristics of the Gaussian distribution are either constant (a) or varying (b) across the 13 signals.

For our real data experiments, we used sub-spectra acquired by probing chip spots at positions ranging from 20 to 80, with intervals of 5 (see Methods section in main text). The simulation experiment suggests that it may be beneficial to probe multiple spot locations, for example by decreasing the interval between shot positions. Of course, when intervals become too small, the correlation between sub-spectra taken at adjacent spot positions increases. This is likely to lead to a situation in which the improvement in detected signal-to-noise ratio reaches a plateau, although in this study we have not investigated this conjecture any further.

2. Illustration of wavelet analysis

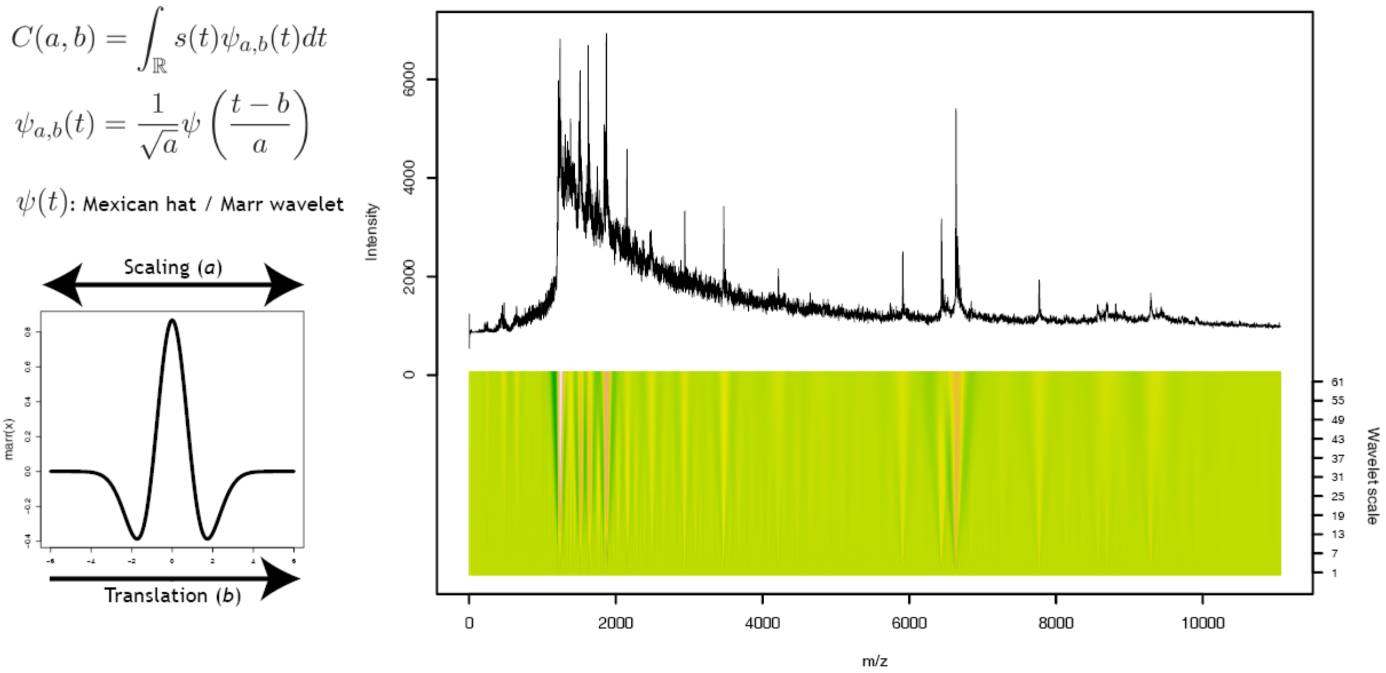

Figure S2: We employ a mexican hat mother wavelet, which is proportional to the second derivative of a Gaussian function and is similar in shape to actual peaks found in a mass spectrum. The mother wavelet is scaled and translated over spectra, during which the inner product between the wavelet and the signal is calculated, resulting in a scale space of wavelet coefficients. This scale space is used to extract features, i.e., peaks, from spectra. Strong ridges in this space are identified, by locating local maxima over multiple scales. These are then filtered according to a certain parameter set, consisting of, among others, the range of scales in which peaks are detected, the minimal length of identified ridges and a signal-to-noise ratio threshold. The m=z axis locations of the ridges that pass these filtering steps are labeled as peak positions.

3. Estimation of ‘peak’ distribution in a virtually empty (matrix only) spectrum

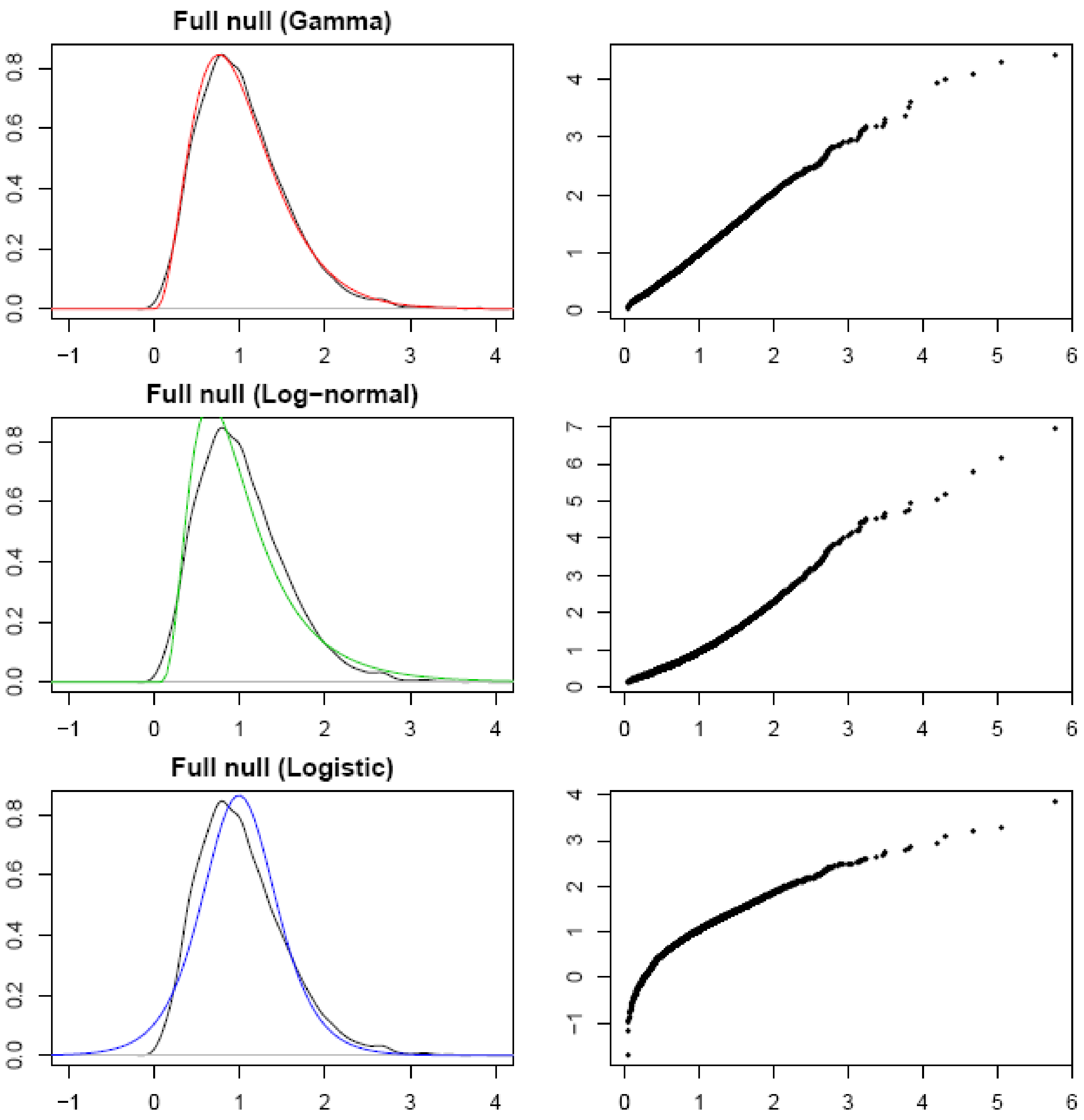

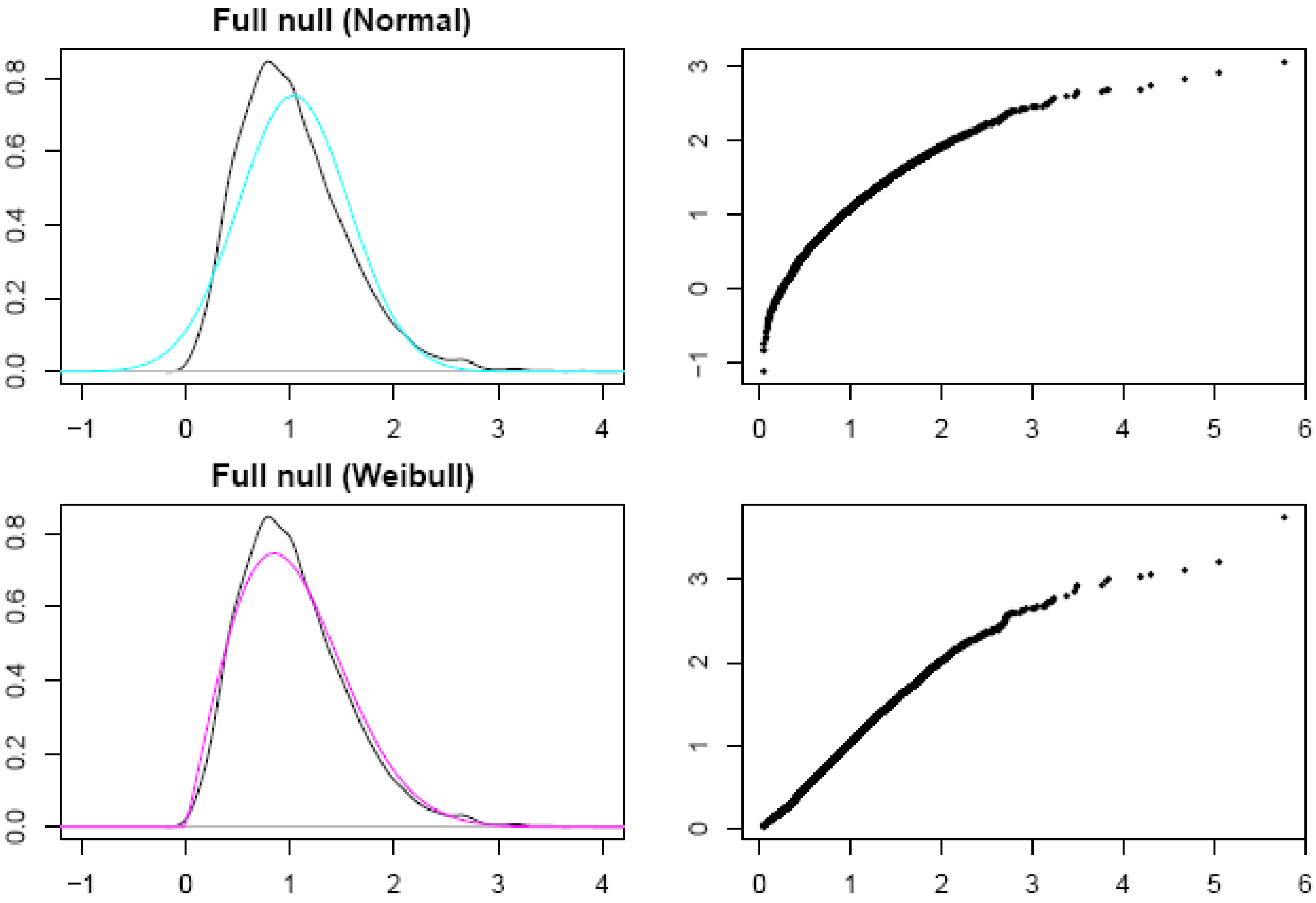

Figure S3: Left panels show distribution of wavelet coefficients for an (almost) empty spectrum (black lines) and different theoretical distributions fitted to it. Right panels show qqplots for these. The Gamma distribution gives the best fit.

4. Estimation of Q from P

Figure S4 shows the maximum squared error between the distribution of wavelet coefficients, from which high values (i.e., real peaks) are iteratively removed and a Gamma distribution of which the parameters were estimated from this data. The x-axis shows the fraction of points included. The red vertical line shows the optimal point; peaks below this point are retained for the 'noise' distribution *Q*.

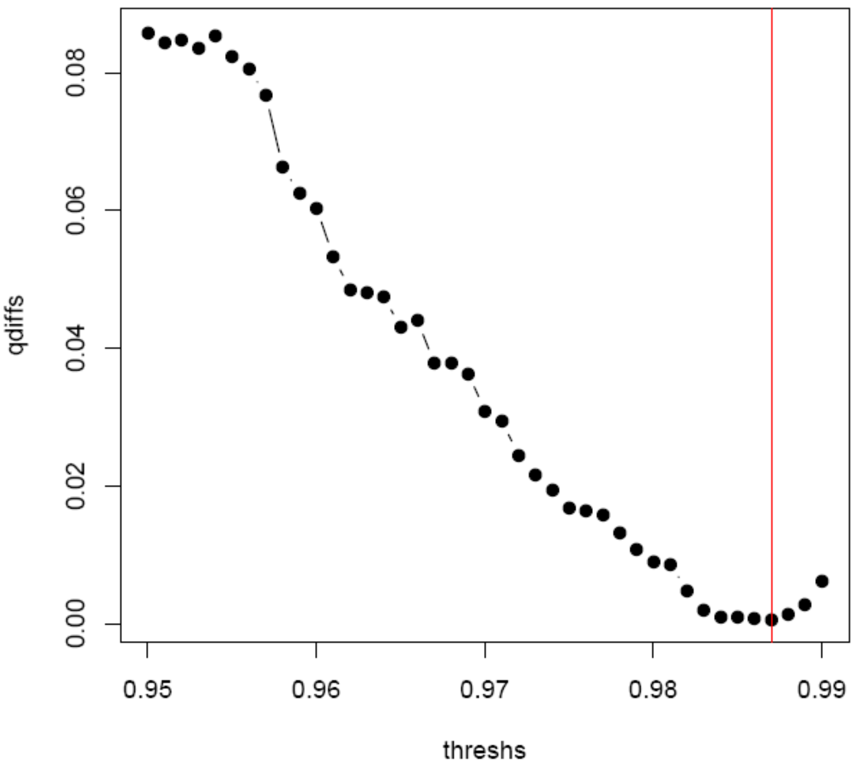

Figure S4: Maximum Squared Error between distribution of wavelet coefficients and Gamma fit.

5. Example of spectrum of spiked human serum

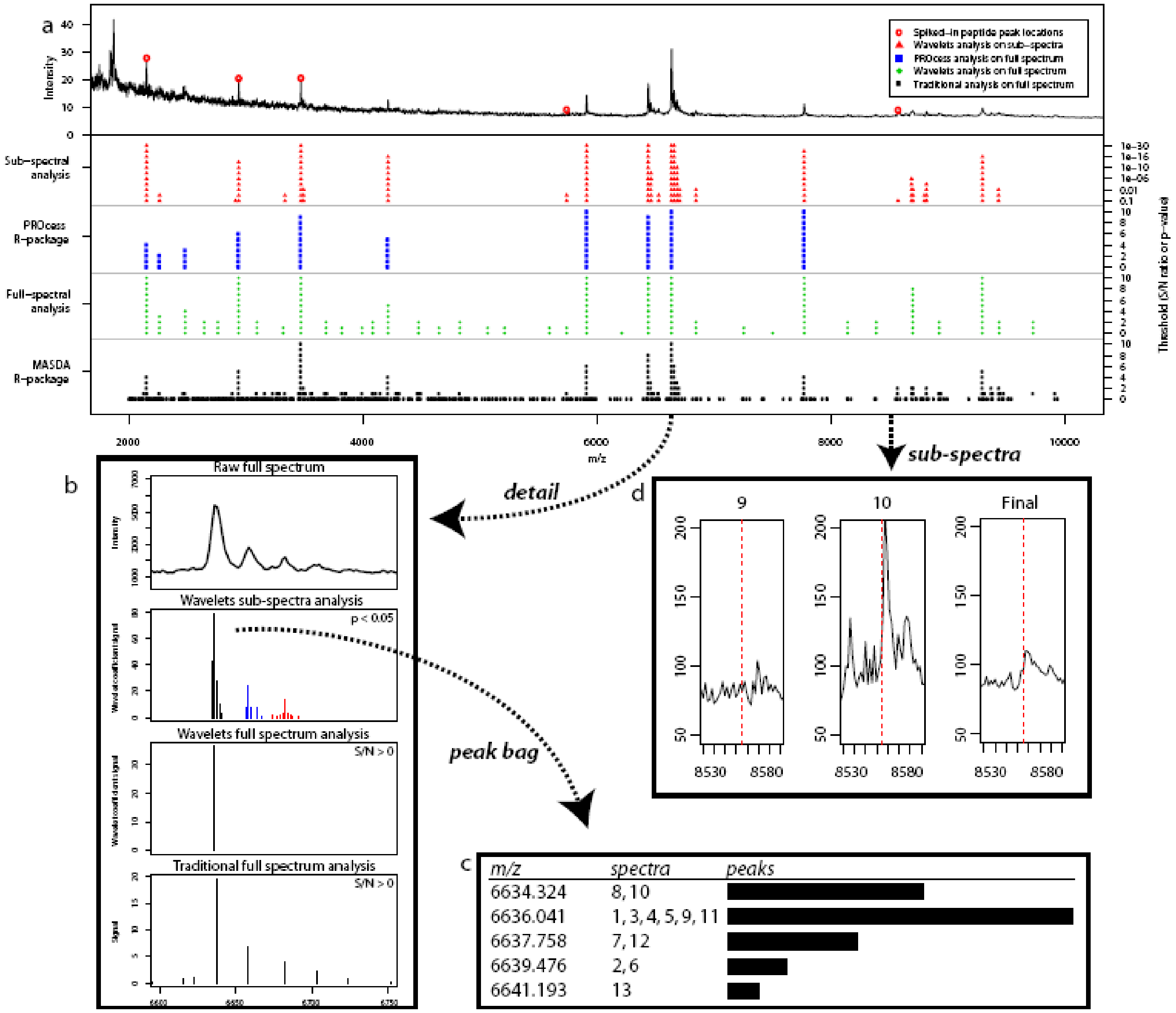

Figure S5: (a) Example spectrum of human serum artificially spiked with 5 peptides of known mass. Open circles (red) on top of peaks, indicate the positions of these peptides in the spectrum. Triangles (red) indicate positions of peaks found using our algorithm. Squares (blue), diamonds (green) and circles (black) show results obtained by analysing the full spectrum using PROcess, wavelets and MASDA, respectively. The latter three employ signal-to-noise thresholds, for a number of which the results are shown. A typical threshold used is 3. (b) Detailed look at the region ranging from m/z between 6600 and 6750. Panels show the full spectrum and results for our algorithm, wavelet analysis on full spectrum and MASDA on full spectrum, respectively. For completeness, a signal-to-noise ratio threshold of 0 is used. (c) Detailed view of the contents of one peak-bag, as detected by our algorithm. Note that all 13 sub-spectra contribute to this particular peak-bag. (d) Example of a spiked peptide which could pose problems when trying to detect it in a full spectrum; much information about variance etc. between sub-spectra is discarded this way. The panels show two sub-spectra (spot positions 9 & 10) and the full spectrum (full), illustrating how peaks can be averaged out in case of large signal differences between sub-spectra.

6. Procedure to obtain sub-spectra

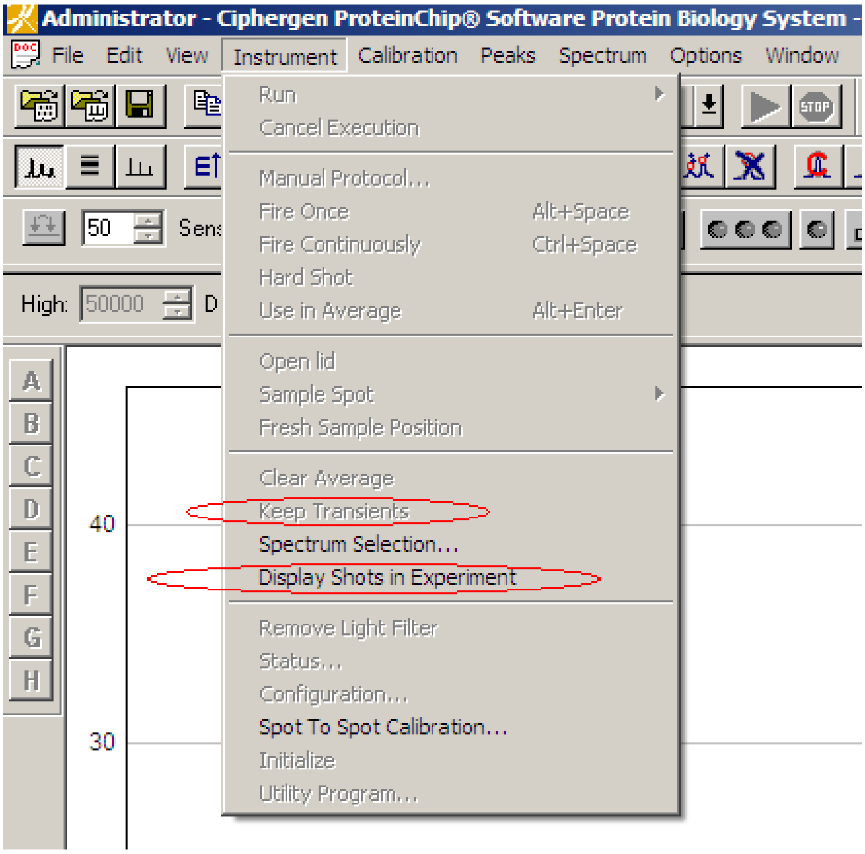

Figure S6: Screenshot of 'Instrument' Menu of Ciphergen ProteinChip© Software version 3.1.

This procedure is for Ciphergen ProteinChip© Software version 3.1

1. Before the chip is analysed, make sure to select 'Keep transients' from the Instrument menu.
2. Then, after the analysis, select 'Display Shots in Experiment' to open up a new window containing all sub-spectra.
